# Cloning and Functional Characterization of *SpZIP2*

**DOI:** 10.3390/genes13122395

**Published:** 2022-12-17

**Authors:** Tian-Long Han, Ting-Wei Tang, Pei-Hong Zhang, Min Liu, Jing Zhao, Jia-Shi Peng, Shuan Meng

**Affiliations:** 1 College of Agronomy, Hunan Agricultural University, Changsha 410128, China; 2 School of Life and Health Sciences, Hunan University of Science and Technology, Xiangtan 411201, China; 3 Xiaoxiang College, Hunan University of Science and Technology, Xiangtan 411201, China; 4 College of Bioscience and Biotechnology, Hunan Agricultural University, Changsha 410128, China; 5 Hunan Provincial Key Laboratory of Rice Stress Biology, Changsha 410128, China

**Keywords:** *S. plumbizincicola*, ZIP2, Cd tolerance, Cd accumulation

## Abstract

Zinc (Zn)-regulated and iron (Fe)-regulated transporter-like proteins (ZIP) are key players involved in the accumulation of cadmium (Cd) and Zn in plants. *Sedum plumbizincicola* X.H. Guo et S.B. Zhou ex L.H. Wu (*S. plumbizincicola*) is a Crassulaceae Cd/Zn hyperaccumulator found in China, but the role of ZIPs in *S. plumbizincicola* remains largely unexplored. Here, we identified 12 members of *ZIP* family genes by transcriptome analysis in *S. plumbizincicola* and cloned the *SpZIP2* gene with functional analysis. The expression of *SpZIP2* in roots was higher than that in the shoots, and Cd stress significantly decreased its expression in the roots but increased its expression in leaves. Protein sequence characteristics and structural analysis showed that the content of alanine and leucine residues in the SpZIP2 sequence was higher than other residues, and several serine, threonine and tyrosine sites can be phosphorylated. Transmembrane domain analysis showed that SpZIP2 has the classic eight transmembrane regions. The evolutionary analysis found that SpZIP2 is closely related to OsZIP2, followed by AtZIP11, OsZIP1 and AtZIP2. Sequence alignment showed that most of the conserved sequences among these members were located in the transmembrane regions. A further metal sensitivity assay using yeast mutant Δ*yap1* showed that the expression of *SpZIP2* increased the sensitivity of the transformants to Cd but failed to change the resistance to Zn. The subsequent ion content determination showed that the expression of *SpZIP2* increased the accumulation of Cd in yeast. Subcellular localization showed that SpZIP2 was localized to membrane systems, including the plasma membrane and endoplasmic reticulum. The above results indicate that *ZIP* member *SpZIP2* participates in the uptake and accumulation of Cd into cells and might contribute to Cd hyperaccumulation in *S. plumbizincicola*.

## 1. Introduction

As a non-essential mineral element in plants, cadmium (Cd) is absorbed and translocated by plants through the transport system of other elements such as Zinc (Zn), manganese (Mn) and iron (Fe), and its presence in the food chain threatens human health [1]. Elucidation of the underlying mechanisms would be of great importance to either reducing heavy metal translocation into food chains or performing phytoremediation of the Cd-polluted soils.

*Sedum plumbizincicola* X.H. Guo et S.B. Zhou ex L.H. Wu (*S. plumbizincicola*) is a Cd/Zn hyperaccumulator found in China that can accumulate high amounts of heavy metals without any obvious physiological toxicity to the plant [2,3,4,5]. Thus, *S. plumbizincicola* is widely used for the effective remediation of Cd-contaminated agricultural soils [6,7,8] and is capable of achieving high remediation efficiency using appropriate agronomic strategies [9,10,11], even in alkaline soil [12] and highly polluted soils [13]. *S. plumbizincicola* also plays an important role in enhancing the fertility of calcareous soil [14] and the mobilization of less active metal fractions [15] during remediation. Moreover, *S. plumbizincicola* can obtain transgenic plants using the agrobacterium-mediated genetic transformation method [16] and thus, it is becoming an important research object to analyze the accumulation and tolerance of Cd in plants. The cell wall and several key genes were identified as key players in the process of Cd hyperaccumulation for *S. plumbizincicola*. The chloroplast-located SpHMA1 is involved in the efflux of Cd from the chloroplast, thereby preventing the accumulation of Cd in the chloroplast to protect photosynthesis [17]. Meanwhile, SpHMA3 is localized to the vacuolar membrane and has a specific ability to transport Cd ions into the vacuole for compartmentalization and realize cytoplasmic detoxification [18]. The overexpression of *SpbZIP60* in *Arabidopsis thaliana* (*A. thaliana*) increases the ability of Cd tolerance in transgenic plants [19]. The overexpression of *SpHMA7* in yeast was found to increase yeast sensitivity to Cd [20], whereas the expression of SpMT2 and MTL in yeast increased its tolerance to Cd [21,22]. Compared with the non-hyperaccumulator *Sedum alfredii (S. alfredii*), the increased expression level and protein sequence variation of MTL in *S. plumbizincicol* is crucial for its Cd hyperaccumulation and hypertolerance [21].

Members from the Zinc (Zn)-regulated and iron (Fe)-regulated transporter-like proteins (ZIP) family can transport metal elements, such as Zn, Mn, Fe and Cd, and display differential substrate transport activity [23,24,25]. A total of 15 ZIP members have been identified in *A. thaliana* [26]. AtIRT1, the first reported ZIP member, is a key component in maintaining iron homeostasis in *A. thaliana* [27,28]. AtZIP1-AtZIP4 can functionally complement Zn-uptake-deficient yeast strains [29]. Additionally, AtZIP1 and AtZIP2 are also involved in the transport of Mn from roots to shoots [30]. In rice, there are 16 ZIP members, including 14 Zn-regulated transporters and 2 Fe-regulated transporters (IRT) [23]. ZIP family members such as OsIRT1 [31,32], OsIRT2 [33], OsZIP1 [34], OsZIP3 [35], OsZIP4 [36], OsZIP5 [37], OsZIP7 [38,39,40], OsZIP8 [38,41] and ZIP9 [42] were reported to transport Zn and/or Fe. ZIP family members have also been found to participate in transmembrane Cd transport. For example, AtIRT1, AtIRT2, OsIRT1, OsIRT2, OsZIP5 and OsZIP9 are involved in the uptake and transport of Cd by rice roots [32,42,43,44,45]. *OsZIP6* mediated Cd uptake when expressed in *Xenopus* oocytes [46]. Expressing *OsZIP1* and *OsZIP3* in yeast could enhance Cd sensitivity and promote Cd accumulation [45]. The co-expression of *OsLCT1-OsHMA2-OsZIP3* in rice effectively reduced Cd accumulation in grains [47]. Moreover, transgenic rice plants with higher expression levels of *OsZIP1* were shown to reduce Cd accumulation and toxicity [34].

There is sufficient evidence to conclude that ZIP members are key players in Cd and Zn accumulation; however, *SpZIPs* in the Cd/Zn hyperaccumulator *S. plumbizincicola* are still unreported. In this study, we identified 12 *ZIP* genes in *Sedum*. Among the higher expressed *ZIP* members, *ZIP2* was the only lower expressed *ZIP* gene in *S. plumbizincicola* than in *S. alfredii*, indicating that *ZIP2* might have a special function in Cd/Zn hyperaccumulation compared with other *ZIPs.* We therefore designed a study to isolate *SpZIP2* and investigate its basic features and transport activity, aiming to provide clues for further uncovering the mechanism of Cd/Zn hyperaccumulation in *S. plumbizincicola*.

## 2. Materials and Methods

### 2.1. Plant Materials and Growth Conditions

*S. plumbizincicola* and *S. alfredii* were collected from a discarded mine (N29.22, E118.78, Huiping town, Zhejiang Province). Young and healthy lateral shoots from plants at a similar growth stage were cut off and grown in non-contaminated soils in phytotron for several generations. Then, young lateral shoots at a similar growth stage were cultivated in hydroponic solution in phytotron at 24–26 °C with 16 h light/8 h dark cycles, as described in [21]. The details of cultivation are as follows: Several big pots were used for cultivating enough *S. plumbizincicola* and *S. alfredii* plants for further experiments. Each pot contained 6 L of nutrient solution and 24 seedlings. The solution was replaced every 2–3 days. After hydroponic cultivation for about 8 weeks, *S. plumbizincicola* and *S. alfredii* plants were treated with either normal hydroponic solution or a solution containing 50 μM Cd for another 3 days using several small pots. Each experimental small pot contained 3 L of nutrient solution and 6 seedlings.

### 2.2. Analyses of Expression Pattern for SpZIP in S. plumbizincicola and S. alfredii

The transcriptome and genome sequencing methods used were described previously [3] and used to obtain FPKM values of genes annotated as ZIPs. Then, a heatmap was constructed using OECloud tools at https://cloud.oebiotech.cn (accessed on 8 August 2022) with default parameters. Quantitative RT-PCR analysis was conducted as follows: 8-week-old *S. plumbizincicola* plants were treated with normal or 50 μM Cd solution for another 3 days, following which roots and shoots were harvested, and gene expression levels were detected. The primer sequences are provided in Table S1.

### 2.3. Sequence Characterization and Transmembrane Region Prediction

The amino acid compositions of SpZIP2 were analyzed by Expasy (https://web.expasy.org/protparam/, accessed on 1 November 2022) with default parameters [48]. Transmembrane domains of SpZIP2 were analyzed using Phobius (http://phobius.sbc.su.se/, accessed on 4 August 2022) [49]. Protein phosphorylation sites of SpZIP2 were predicted using NetPhos-3.1 (https://services.healthtech.dtu.dk/service.php? NetPhos-3.1, accessed on 14 July 2022) [50].

### 2.4. Phylogenetic Analysis and Sequence Alignment

The amino acid sequences of ZIPs from *A. thaliana* and rice were downloaded from the websites using gene accession numbers as below: AtIRT1(AT4G19690.2), AtIRT2(AT4G19680.2), AtIRT3(AT1G60960.1), AtZIP1(AT3G12750.1), AtZIP2(AT5G59520.1), AtZIP3(AT2G32270.1), AtZIP4(AT1G10970.1), AtZIP5(AT1G05300.1), AtZIP6(AT2G30080.1), AtZIP7(AT2G04032.1), AtZIP8(AAL83293.1, The European Nucleotide Archive), AtZIP9(AT4G33020.2), AtZIP10(AT1G31260.1), AtZIP11(AT1G55910.1), AtZIP12(AT5G62160.1), OsIRT1(LOC_Os03g46470.1), OsIRT2(LOC_Os03g46454.1), OsZIP1(LOC_Os01g74110.1), OsZIP2(LOC_Os03g29850.1), OsZIP3(LOC_Os04g52310.1), OsZIP4(LOC_Os08g10630.1), OsZIP5(LOC_Os05g39560.1), OsZIP6(LOC_Os05g07210.1), OsZIP7(LOC_Os05g10940.1), OsZIP8(LOC_Os07g12890.1), OsZIP9(LOC_Os05g39540.1), OsZIP10(LOC_Os06g37010.1), OsZIP11(LOC_Os05g25194.1), OsZIP13(LOC_Os02g10230.1), OsZIP14(LOC_Os08g36420.3) and OsZIP16(LOC_Os08g01030.1). Sequence alignment analysis was performed using CLUSTALW (https://www.genome.jp/tools-bin/clustalw, accessed on 8 August 2022) with default parameters, and the results were displayed by ESPript3.x (https://espript.ibcp.fr/ESPript/cgi-bin/ESPript.cgi, accessed on 8 August 2022) [51]. The phylogenetic tree was constructed online (https://ngphylogeny.fr/, accessed on 8 August 2022) with default parameters [52] and then displayed using the iTOL tool (https://itol.embl.de/, accessed on 8 August 2022) [53].

### 2.5. Cd Tolerance and Accumulation Analyses Using Yeast Strain

The CDS region of *SpZIP2* was amplified with primers *SpZIP2*-BL and *SpZIP2*-ER (Table S1) using *S. plumbizincicola* cDNA as the template and inserted into vector pYES2 with BamH1 and EcoR1 restriction sites to generate pYES2-*SpZIP2*. Empty vector pYES2 and the constructed pYES2-*SpZIP2* vector were each transformed into yeast Δ*yap1*, and the empty vector pYES2 was also transformed into wild-type yeast Y252 as described in [54]. The correct clone was selected to be grown in liquid to log phase for further analysis.

Metal sensitivity assay: The cultures were gradient diluted as indicated, dropped onto SD plates containing different Cd concentrations and then grown at 30 °C for about 7 days before being photographed.

Metal accumulation assay: Yeast cells were cultured to log phase in liquid SD medium and then allowed to grow for another 6 h in liquid SD medium with 50 μM CdCl_2_. Yeast cells were collected, washed and dried, and then the Cd content in the cells was determined by ICP-MS as described [21].

### 2.6. Subcellular Localization Analyses

The CDS region of *SpZIP2* was amplified with primers *SpZIP2*-BL and *SpZIP2*-E-R2 (Table S1) and then inserted into the pYES2-*mRFP* vector using restriction sites BamH1 and EcoR1 to generate pYES2-*mRFP*-*SpZIP2*. Vectors pYES2-*mRFP* and pYES2-*mRFP*-*SpZIP2* were each transformed into yeast Δ*yap1*. The correct clone was identified and cultured to log phase in liquid SD medium. Fluorescence was observed by a confocal laser microscope. A Cd sensitivity assay was carried out according to the method described in Section 3.4. Subcellular localization of SpZIP2 in plants was predicted by Plant-mPLoc [55] with default parameters.

### 2.7. Statistical Analyses

Statistical significance was tested by two-tailed Student’s *t*-tests using Microsoft Excel 2010 (Version number is 14.0.7268.5000, and was sourced from Changsha, China). Differences were deemed significant at *p* < 0.05 (*).

## 3. Results

### 3.1. Expression Pattern of SpZIP2

**Figure 1:**
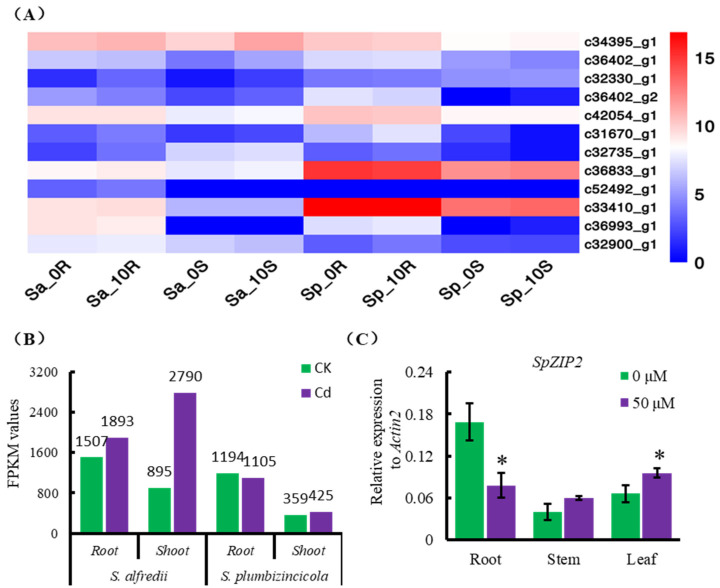
Expression pattern of Zinc (Zn)-regulated and iron (Fe)-regulated transporter-like proteins *(ZIPs)* in *Sedum plumbizincicola (S. plumbizincicola*) and *Sedum. alfredii (S. alfredii*). (**A**) Expression of *ZIP* genes in *S. plumbizincicola* and *S. alfredii* in response to Cd stress. Sp: *S. plumbizincicola*. Sa: *S. alfredii*. 0R: Roots without Cd treatment. 10R: Roots with Cd treatment. 0S: Shoots without Cd treatment. 10S: Shoots with Cd treatment. *ZIP1*: *c33410_g1*; *ZIP2*: *c34395_g1*; *ZIP3*: *c31670_g1*; *ZIP4*: *c36833_g1*. (**B**) FPKM values of *ZIP2* in roots and shoots under normal or Cd stress conditions in *S. plumbizincicola* and *S. alfredii*. (**C**) Expression of *SpZIP2* in roots, stems and leaves in response to Cd treatment in *S. plumbizincicola*. Values are means ± SD, *n* = 3. Statistical significance was tested by Student’s *t*-tests. Differences were deemed significant at *p* < 0.05 (*).

We analyzed the genes’ response to Cd treatment in *S. plumbizincicola* and *S. alfredii* by combining comparative transcriptomic and genomic sequencing obtained previously [3]. The results revealed that the expressions of *ZIP* family genes *SpZIP1* (*c33410_g1*), *SpZIP3* (*c31670_g1*) and *SpZIP4* (*c36833_g1*) were much higher in *S. plumbizincicola* than those in *S. alfredii* [3]. Further analysis identified 12 *ZIP* family members in *S. plumbizincicola* and *S. alfredii*. In *S. plumbizincicola,* the expression level of *SpZIP2* was higher in roots than in shoots, and both were lower than *SpZIP1* and *SpZIP4* but higher than *SpZIP3* (Figure 1A). Interestingly, the expression level of *ZIP2* (*c34395_g1*) in *S. alfredii* was higher than that in *S. plumbizincicola* under normal conditions, and this difference was enlarged after Cd treatment (Figure 1B). Quantitative RT-PCR results indicated that the expression of *SpZIP2* was repressed in roots and induced in leaves in response to Cd treatment (Figure 1C).

### 3.2. Structure and Characteristics of SpZIP2

**Figure 2:**
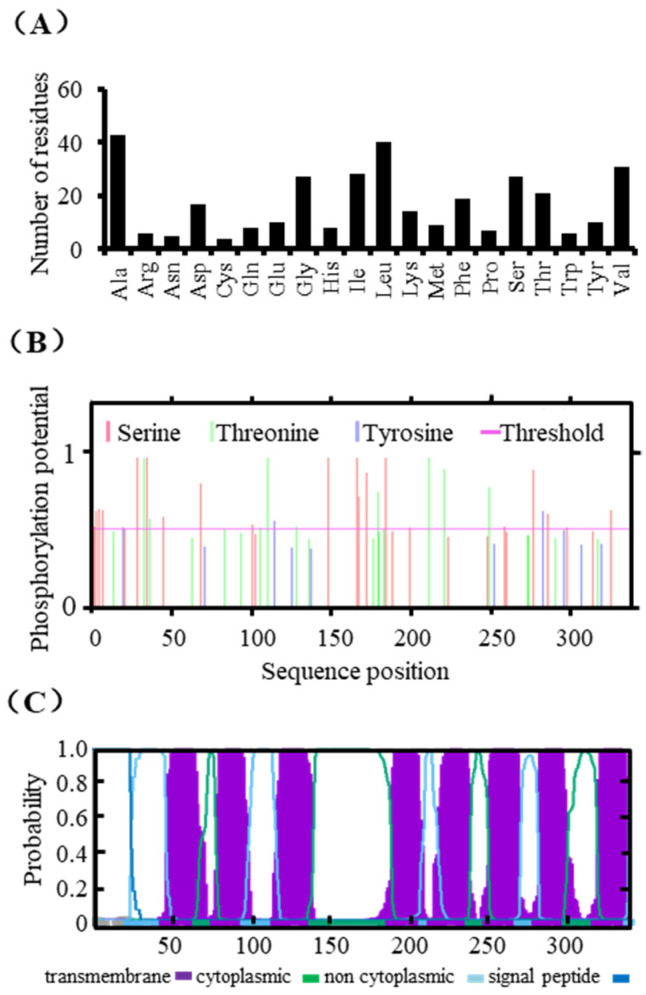
Sequence characterization and transmembrane region prediction of SpZIP2. (**A**) Amino acid composition analysis for SpZIP2. (**B**) Prediction of protein phosphorylation sites for SpZIP2. (**C**) Transmembrane region prediction of SpZIP2.

Based on the transcriptome and genome sequencing, we obtained the coding sequences and amino acid sequences of the *SpZIPs* (Table S2). The basic characteristics of SpZIP2 were further analyzed using bioinformatics methods. Amino acid composition analysis showed that SpZIP2 consisted of 340 amino acids, among which the proportion of alanine residues was the highest at 12.6%, followed by leucine at 11.8%; the two least-contented amino acids residues were cysteine and asparagine, accounting for 1.2% and 1.5%, respectively (Figure 2A). Phosphorylation site prediction analysis showed that SpZIP2 had 31 possible sites that could be phosphorylated, including 19 serine sites, 9 threonine sites and 3 tyrosine sites (Figure 2B). Transmembrane domain analysis indicated that SpZIP2 has eight potential transmembrane domains with a long variable region between the third and fourth transmembrane regions (Figure 2C), which is consistent with the other ZIP family members [25].

### 3.3. Phylogenetic Analyses and Sequence Alignment of SpZIP2 with Other ZIP Members

**Figure 3:**
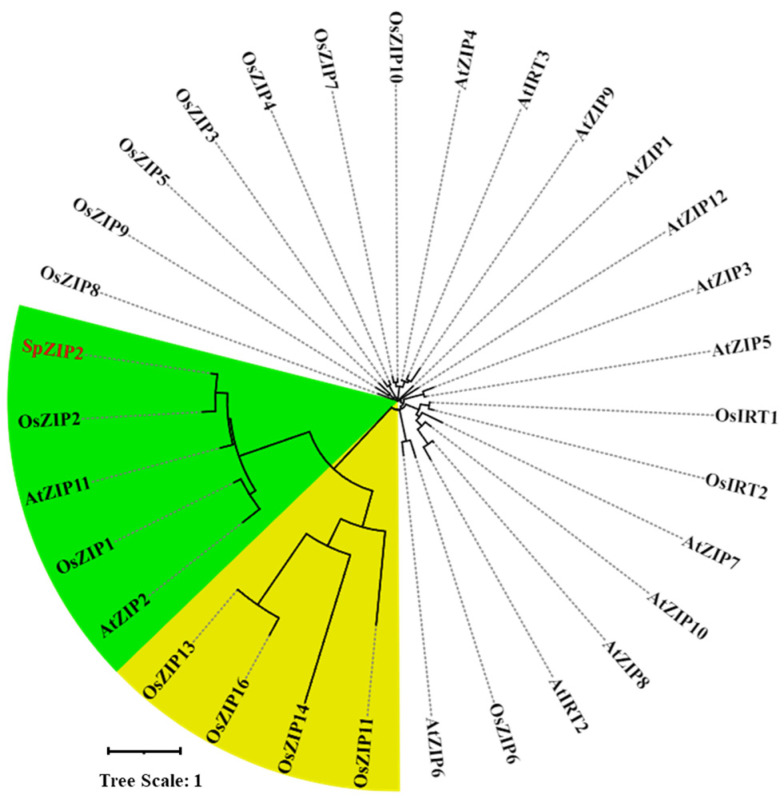
Phylogenetic analysis for SpZIP2 and ZIP members from rice and *Arabidopsis thaliana* (*A. thaliana*). SpZIP2 is shown in red color and its closely related ZIP members are shown by a green background.

We further obtained rice and *A. thaliana* ZIP family protein sequences and performed an evolutionary analysis with SpZIP2. The results showed that SpZIP2 is more closely related to OsZIP1 and OsZIP2 in rice and AtZIP11 and AtZIP2 in *A. thaliana* (Figure 3), suggesting that these members might have similar functions.

**Figure 4:**
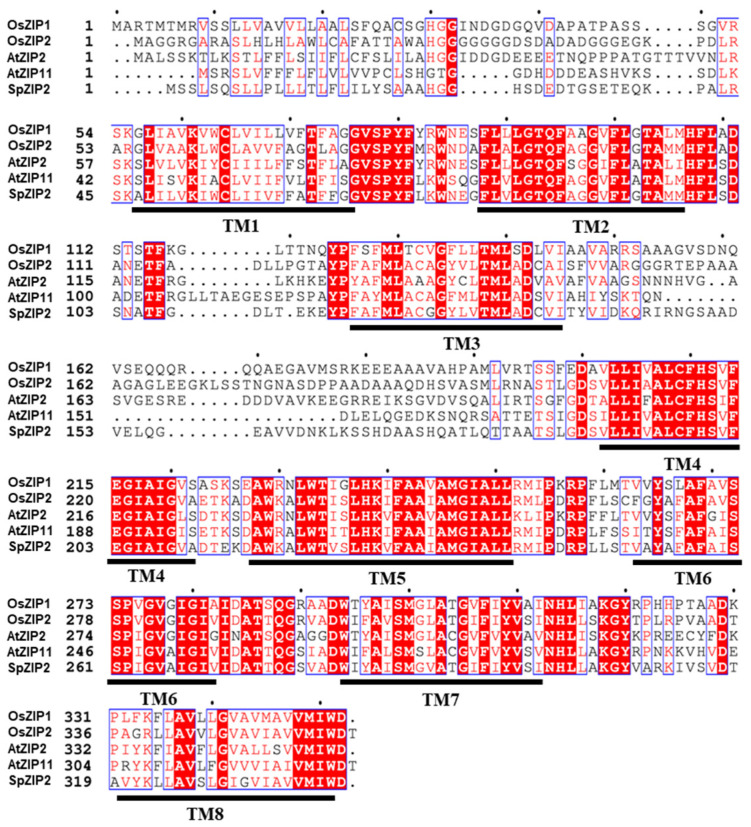
Sequence alignment of SpZIP2 and ZIP members from rice and *A. thaliana*. Strictly conserved residues are highlighted in red. The corresponding positions of the predicted 8 transmembrane regions are indicated as TM1 to TM8 below the corresponding sequence.

We then performed sequence alignment analysis with SpZIP2 and its closely related members OsZIP1, OsZIP2, AtZIP11 and AtZIP2. These members have low sequence similarity between the N-terminal and the regions between the third and fourth transmembrane domains. Meanwhile, most of the highly conserved sequences are located in the transmembrane region (Figure 4).

### 3.4. SpZIP2-Mediated Cd Tolerance and Accumulation in Yeast

**Figure 5:**
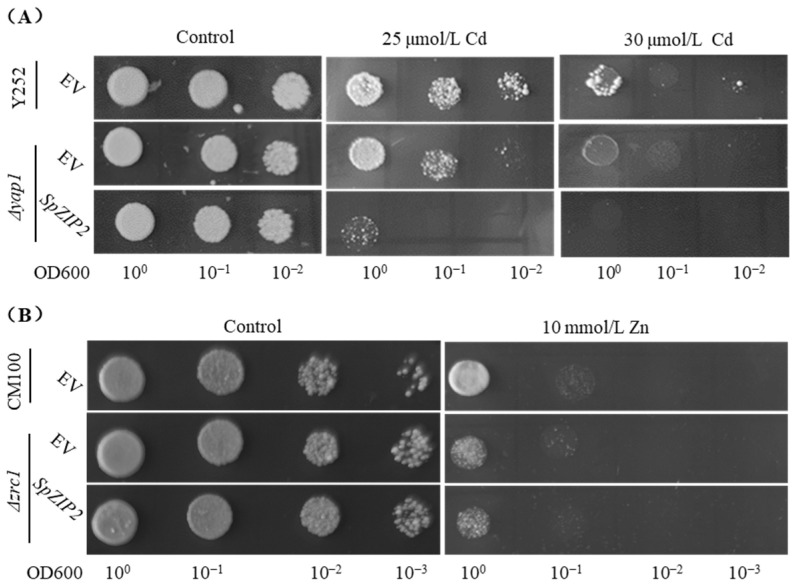
Tolerance of yeast transformants expressing *SpZIP2* to Cd or Zn stresses. (**A**) Tolerance of yeast transformants expressing *SpZIP2* to Cd stress. (**B**) Tolerance of yeast transformants expressing *SpZIP2* to Zn stress.

Given that *S. plumbizincicola* is a Cd/Zn hyperaccumulator plant, we then analyzed the roles of *SpZIP2* for Cd and Zn tolerance in the heavy-metal-sensitive yeast mutant Δ*yap1*. The yeast Δ*yap1* expressing *SpZIP2* hardly grew under external 25 μM Cd treatment, while theΔ*yap1* transformed with an empty vector showed a more effective growth status (Figure 5A), indicating that the yeast Δ*yap1* expressing *SpZIP2* was more sensitive to Cd. Notably, there was no significant growth difference between pYES2-*SpZIP2* and empty vector pYES2 transformants under external Zn treatment conditions (Figure 5B). The above results indicate that *SpZIP2* expression in yeast specifically improved the sensitivity of yeast to Cd.

**Figure 6:**
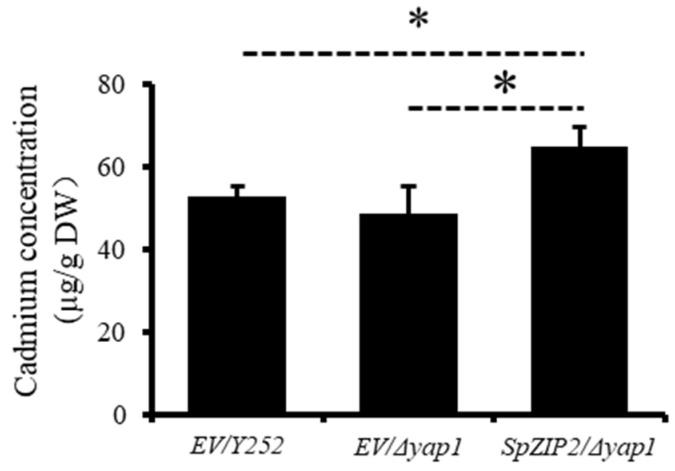
Cd accumulation in *SpZIP2* expressed *Δyap1* and empty vector transferred yeast Y252 and *Δyap1*. Values are means ± SD, *n* = 3. Statistical significance was tested by Student’s *t*-tests. Differences were deemed significant at *p* < 0.05 (*).

To further uncover the details of *SpZIP2* in increasing Cd sensitivity in yeast, we measured the Cd content in yeast. As shown in Figure 6, the Cd content in yeast Δ*yap1* expressing *SpZIP2* reached 64.92 μg/g (dry weight), while the Cd content in yeast transformed with the empty vector was only 48.46 μg/g yeast dry weight, which was much lower than the former. This indicates that expression of *SpZIP2* in yeast can greatly increase the Cd content, and SpZIP2 could effectively uptake external Cd into yeast cells.

### 3.5. SpZIP2 Localized to Membrane Systems Include Plasma Membrane

**Figure 7:**
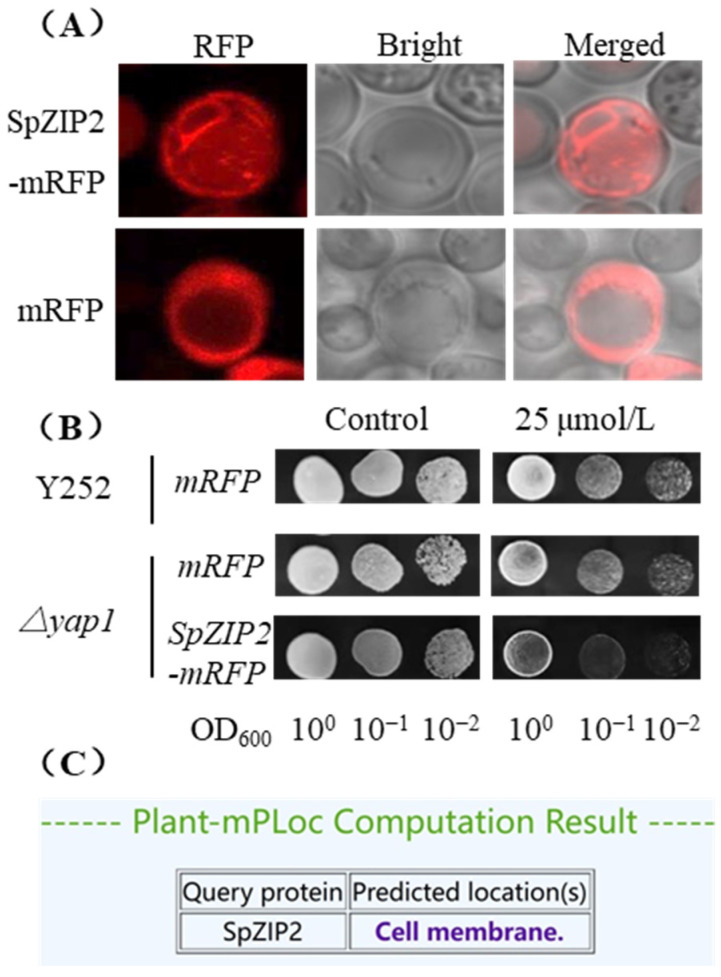
Subcellular localization analysis of SpZIP2. (**A**) Subcellular localization of SpZIP2-mRFP fusion proteins or mRFP in yeast Δ*yap1.* (**B**) Tolerance of yeast transformants expressing SpZIP2-mRFP to Cd stress. (**C**) Subcellular localization prediction for SpZIP2 by Plant-mPLoc.

Expression of *SpZIP2* led to more sensitivity to Cd and increased the Cd content in yeast, indicating that it was likely to mediate the transmembrane transport of Cd across the yeast plasma membrane. We then constructed the pYES2-*SpZIP2*-*mRFP* vector and performed subcellular localization analysis. The results showed that the fluorescence of SpZIP2-mRFP was detected in multiple membrane systems, including the plasma membrane (Figure 7A). SpZIP2-mRFP fusion was proven functional by a Cd sensitivity assay which showed that yeast expressing the SpZIP2-mRFP fusion protein exhibited a Cd-sensitive phenotype compared to yeast expressing mRFP alone (Figure 7B). Those results, together with the results from Figure 5 and Figure 6, suggest that SpZIP2 is involved in the uptake of external Cd in yeast. Notably, a subcellular localization prediction analysis for plant proteins indicated that SpZIP2 is a plasma-membrane-localized protein (Figure 7C).

## 4. Discussion

Members of the *ZIP* family play an important role in the uptake and accumulation of metals in plants. ZIP family members have been functionally identified in many species, including *A. thaliana* and rice. Through genome-wide identification and analysis, 12, 12, 21, 20, 30, 33 and 14 ZIP family members were identified in maize, potato, poplar, lettuce, peanut, wild emmer wheat and hexaploid wheat, respectively [43,56,57,58,59,60,61,62]. Based on transcriptome and genome sequencing, we identified 12 ZIP members in *S. plumbizincicola* and *S. alfredii*, among which the expression levels of *ZIP1*, *ZIP3* and *ZIP4* were much higher in *S. plumbizincicola* than those in *S. alfredii*. The expression levels of *ZIP1*, *ZIP3* and *ZIP4* in the roots of *S. plumbizincicola* are much higher than those in the shoots (Figure 1A). Given that the roots are responsible for Cd uptake from the soil and its translocation to shoots [1], these three members may therefore play a role in the above-mentioned process. However, the accurate function of these three members requires further evidence, including ion transport activity and tissue/subcellular localization data. Interestingly, the expression level of *ZIP2* was lower in *S. plumbizincicola* than that in *S. alfredii* and was the highest expressed *ZIP* member in *S. alfredii* (Figure 1), suggesting that the gene may play a unique function.

*S. plumbizincicola* is a Cd/Zn hyperaccumulator, and the *ZIP* gene is widely involved in the Cd/Zn transport process in plants [25]. Heterologous expression of *SpZIP2* in yeast effectively mediated the transport of Cd and increased Cd accumulation in yeast (Figure 5 and Figure 6). Previous studies have shown that *OsZIP1* and *OsZIP3* could increase Cd sensitivity and accumulation when expressed in yeast cells [45]. These genes might mediate Cd uptake into cells.

Members from the ZIP family could transport Cd, Mn, Fe, Zn and other elements [23,25] and different members show differential transport activities for different substrates, which is dependent on the sequence specificity between the third and fourth transmembrane segments [25]. SpZIP2 is closely related to OsZIP1, OsZIP2, AtZIP11 and AtZIP2, and the highly similar sequences between those members are mostly located in the transmembrane region, while the similarity between the third and fourth transmembrane regions is very low (Figure 4), which might lead to differences in their substrate transport activity. SpZIP2 may mediate the transport of Cd, but not Zn (Figure 5 and Figure 6); AtZIP2 can transport Zn and Mn, but not Cu and Fe [30]; OsZIP1 can mediate the efflux of Cd, Zn and Cu [34].

The subcellular localization of SpZIP2 in yeast involves multiple membrane systems, including the plasma membrane and the endoplasmic reticulum, suggesting functional diversity. OsZIP1, which is closely related to SpZIP2, is localized to the endoplasmic reticulum and plasma membrane [34], while another closely related member, AtZIP2, is localized to the plasma membrane [30]. Based on the results from Figure 5, Figure 6 and Figure 7, we speculate that *SpZIP2* may be involved in cell Cd influx in *S. plumbizincicola*.

The accumulation of Cd by plants involves several key physiological processes, including the absorption of external Cd by roots and its subsequent transport from roots to shoots [1]. These processes need several key members, among which the absorption of external Cd by roots is mainly controlled by the plasma-membrane-localized protein Nramp5 [63,64], while the process of root transport to the shoot mainly depends on the tonoplast-localized P_1B_-type heavy metal ATPase HMA3, which reduces the transfer of Cd to the shoots by compartmentalizing it to the vacuoles [65,66]. Gene expression results showed that the expression of *ZIP2* in the roots of *S. plumbizincicola* was inhibited by Cd, but was induced in *S. alfredii*, and the expression level was higher in *S. alfredii* than that in *S. plumbizincicola*. Given that *S. plumbizincicola* is a hyperaccumulator and *S. alfredii* is a non-hyperaccumulating control [3,16], it was suggested that *SpZIP2* might not be a main participant in the process of absorbing Cd from the outside. Considering that SpZIP2 functions to uptake Cd into cells, it might play a role in the process of unloading Cd from vascular tissues when expressed in roots, thereby reducing the Cd transfer to the shoots.

However, the clear role of *SpZIP2* in plants needs to be further elucidated in combination with tissue localization analysis and plant genetic materials.

## 5. Conclusions

Using transcriptome analysis, we isolated *SpZIP2* from *S. plumbizincicola.* SpZIP2, with a structure and characteristics similar to ZIP members, could enhance the accumulation of Cd in yeast, thus leading to greater Cd sensitivity. Therefore, *SpZIP2* contributes to the uptake and accumulation of Cd into cells and might participate in Cd hyperaccumulation in *S. plumbizincicola*.

## Data Availability

Not applicable.

## Supplementary Materials

The following supporting information can be downloaded at: https://www.mdpi.com/article/10.3390/genes13122395/s1, Table S1: List of primer sequences used in this study; Table S2: Coding sequence and amino acid composition of SpZIP2.
