# Supplementary material for: Cloning and Functional Characterization of SpZIP2
Source: Genes (Basel). 2022 Dec 17;13(12):2395. doi: 10.3390/genes13122395 (PMC9778510; doi:10.3390/genes13122395)
Supplement: Supplementary file 1 [file genes-13-02395-s001.zip › Table S2.pdf]

Table S2. Coding sequence and amino acid composition of SpZIP2

SpZIP2	Sequences
Coding sequence	ATGTCATCCCTCTCTCAGTCTCTACTCCCACTCCTCCTAACCCTCTTCCTCATC<br>CTCTACTCTGCCGCCGCTCACGGAGGCCACAGCGACGAAGACACAGGCTC<br>AGAAACAGAGCAAAAACCTGCTCTACGTTCAAAAGCGCTAATTCTTGTAAA<br>GATCTGGTGTTTAATCATTGTCTTTTTCGCCACTTTCTTCGGCGGTGTCTCTC<br>CGTACTTCCTTAAATGGAACGAGGGGTTTCTTGTCCTCGGCACGCAGTTTG<br>CTGGCGGCGTGTTTCTCGGAACGGCGATGATGCATTTTTTAAGTGATTCGA<br>ATGCGACTTTTGGGGATTTGACGGAGAAGGAGTATCCGTTCGCTTTCATGT<br>TAGCCTGCGGTGGGTATTTAGTGACCATGTTGGCCGATTGCGTCATCACTTA<br>CGTCATTGATAAACAGAGGATTCGGAATGGCTCTGCTGCCGATGTTGAGCT<br>GCAAGGCGAAGCTGTGGTGGATAACAAGTTGAAGTCAAGCCATGATGCAG<br>CATCTCATCAGGCAACTCTTCAAACAACCGCAGCTACTTCTCTAGGAGACA<br>GTGTACTTCTAATCGTAGCCTTATGTTTCCACTCAGTATTCGAAGGCATCGCC<br>ATCGGTGTTGCCGACACCGAAAAAGACGCATGGAAAGCGCTGTGGACAGT<br>CAGCTTACACAAGGTGTTTGCAGCAATAGCTATGGGCATTGCTCTTCTCAGA<br>ATGATACCAGATCGGCCATTGCTATCAACCGTGGCCTACGCATTTGCTTTCG<br>CCATTTCAAGTCCGATCGGGGTGGCTATCGGGATTGTGATCGATGCAACGA<br>CGCAAGGGTCGGTTGCGGATTGGATTTATGCTATTTCGATGGGAGTGGCTA<br>CTGGGATTTTTATCTATGTTTCGATTAACCATCTGTTGGCAAAGGGGTATGTA<br>GCTCGAAAGATAGTTTCTGTGGACACGGCGGTTTATAAGCTTCTGGCTGTG<br>AGTTTGGGTATTGGGGTTATTGCTGTGGTCATGATATGGGACTGA
Amino acid sequence	MSSLSQSLLPLLLTLFLILYSAAAHGGHSDEDTGSETEQKPALRSKALILVKIWCLI<br>IVFFATFFGGVSPYFLKWNEGFLVLGTQFAGGVFLGTAMMHFLSDSNATFGDL<br>TEKEYPFAFMLACGGYLVTMLADCVITYVIDKQRIRNGSAADVELQGEAVVD<br>NKLKSSHDAASHQATLQTTAATSLGDSVLLIVALCFHSVFEGIAIGVADTEKDA<br>WKALWTVSLHKVFAAIAMGIALLRMIPDRPLLSTVAYAFAFAISSPIGVAIGIVI<br>DATTQGSVADWIYAISMGVATGIFIYVSINHLLAKGYVARKIVSVDTAVYKLLAV<br>SLGIGVIAVVMIWD
