# Supplementary material for: Cloning and Functional Characterization of SpZIP2
Source: Genes (Basel). 2022 Dec 17;13(12):2395. doi: 10.3390/genes13122395 (PMC9778510; doi:10.3390/genes13122395)
Supplement: Supplementary file 1 [file genes-13-02395-s001.zip › Table S1.pdf]

Table S1 List of primer sequences used in this study

Primer name	Primer Sequences (5′–3′)
SpZIP2-B-L	cgggatccATGTCATCCCTCTCTCAGT
SpZIP2-E-R	cggaattcTCAGTCCCATATCATGACC
SpZIP2-E-R2	gggatcctcctcctcctcctcctccGTCCCATATCATGACCAC
SpZIP2-qRT-F	TGCAGCATCTCATCAGGCAA
SpZIP2-qRT-R	GCGCTTTCCATGCGTCTTTT
SpActin-qRT-F	ATGTTCCCTGGTATTGCTGACCGT
SpActin-qRT-R	TCCACATCTGCTGGAAGGTGCTTA

Restriction endonucleases sites were underlined
